# Scald Burns While Bathing Among Elderly People in Japan

**DOI:** 10.7759/cureus.19842

**Published:** 2021-11-23

**Authors:** Rui Suzuki, Hiroyuki Hashimoto, Osamu Okamoto

**Affiliations:** 1 Plastic and Reconstructive Surgery, Beppu Medical Center, Beppu, JPN; 2 Plastic and Reconstructive Surgery, Oita City Medical Association’s Almeida Memorial Hospital, Oita, JPN; 3 Dermatology, Oita City Medical Association’s Almeida Memorial Hospital, Oita, JPN

**Keywords:** elderly, social factors, individual factors, comfortable temperature, low-temperature burn

## Abstract

Night baths are an essential and beloved tradition in Japanese households. The main purpose of taking a bath at the end of the day, besides hygiene, is relaxation. The aging population is rapidly growing in Japan, as one in three people are approaching the age of 65 years or older. Furthermore, with the progress of nuclear families, the number of households with only elderly people and the need for elderly care is increasing. In recent years, elderly people experienced burns caused by hot water during bathing. We report two cases of water bath burns experienced by elderly people.

The first is a case of a 68-year-old woman who presented with a history of type 2 diabetes and osteoarthritis in both knees. She did not notice that the bath stopper was unplugged while she was taking a bath, and she added hot water at around 44°C. She was exposed to hot fluid on the right foot and suffered deep dermal burns. However, due to knee osteoarthritis, it became difficult for her to move. Two hours after taking a bath, she was removed from the bathtub.

The second is a case of a 71-year-old woman who presented with a history of type 2 diabetes and osteoarthritis in both knees. Because the temperature of the bath was approximately 44°C, she tried to cool down the water, but it was difficult for her to move because of knee osteoarthritis. She called out for help from her family living in the neighborhood, but she could not get out. She was sitting for about 2 hours before being noticed by her family. As a result, she suffered second-degree burns on both the buttocks and soles of her feet.

Prolonged exposure to thermal liquids and burns such as low-temperature burns are caused by individual factors, such as decreased perception, orthopedic disease, and difficulty in moving due to fainting, and social factors such as delay in discovery in elderly people living alone. These factors lead to an increased depth of the burn.

## Introduction

Low-temperature burns are caused by prolonged contact with low-temperature heat sources that do not cause injury with short-duration exposure. Temperature-time curves and individual conditions interact to increase the severity of a burn [1]. In fact, there have been reports of burns related to hot-water bottles and heated toilet seat covers [1,2]. Recently, there has been an increase in patients with severe burns due to comfortable-temperature liquids during bathing. Bathing in hot springs and bathtubs is part of Japanese culture, usually with temperatures of approximately 40-45°C. An alarm usually alerts the bather when the temperature rises above a certain level, such as above 50°C. However, comfortable-temperature burns may occur in elderly people and individuals with a history of epilepsy, cerebral infarction, and knee osteoarthritis. Vagal reflexes can occur in response to temperature changes, dehydration, epileptic seizure induction, and physical problems. This reflex may prolong contact with the liquid since the person is unable or unaware of the need to get out of the bath. Unlike a hot-water burn caused by limited contact with a liquid at approximately 100°C, the damage pattern is representative of a low-temperature burn that occurs with the temperature-time curve. As a result, the individual suffers deep burns [3].

Therefore, we propose the term “comfortable-temperature scald burns.” This report presents cases of patients with comfortable-temperature scald burns and points for its medical treatment.

## Case presentation

Case 1

**Figure 1:**
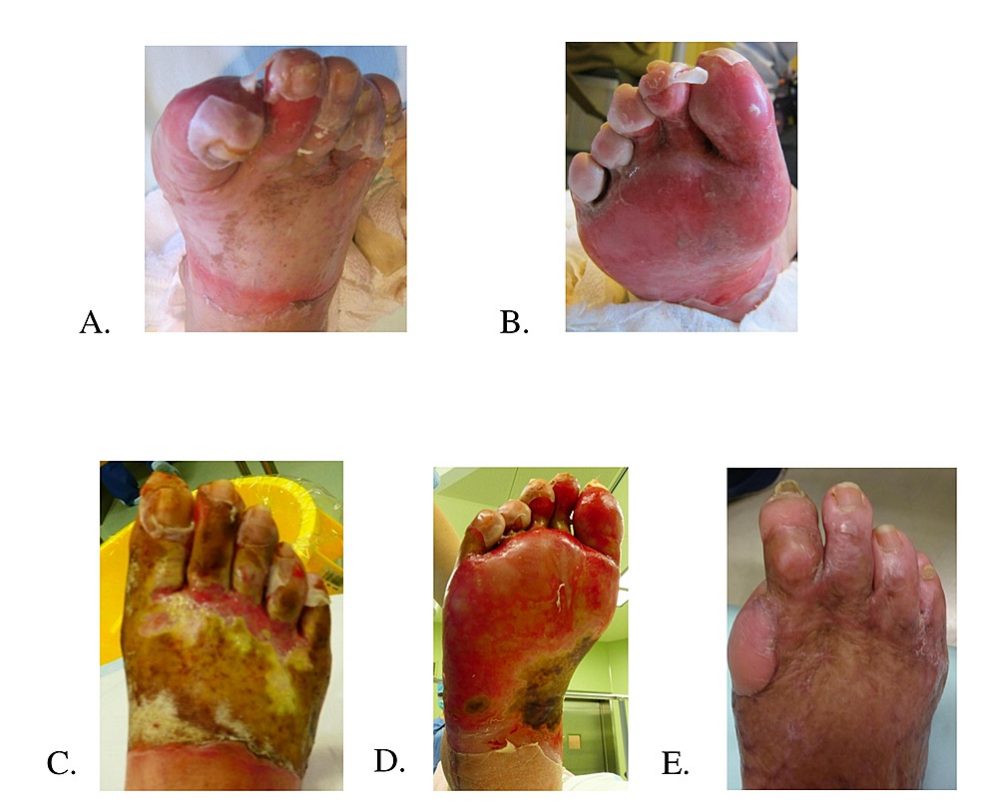
Case 1 (68-year-old woman). (A,B) Day 7 after the burn injury. Concentrated hot fluid exposure from the center to the distal part of the dorsum of the foot. (C,D) Day 10 after the burn injury. White necrotic tissue is found in the arch and dorsum of the foot, and toes except for some parts. (E) Six months after surgery. On the 10th day after the injury, stump plasty was performed on the metatarsophalangeal joint of the toe and debridement and split-thickness skin grafting were performed under general anesthesia. There was no residual ulceration, and the color tone of the skin implantation fragments was almost normal. No hypertrophic scarring was observed.

A 68-year-old woman presented with a history of type 2 diabetes and osteoarthritis in both knees. She did not notice that the bath stopper was unplugged while she was taking a bath, and she added fairly hot water at around 44°C. She was exposed to hot fluid on the right foot and suffered deep dermal burns (Figure 1A, 1B). Due to knee osteoarthritis, it was difficult for her to move the injured foot. She was exposed to the hot liquid for roughly 2 hours before the landlord came home and rescued her from the tub. After the emergency call, the family cooled the patient with cold water for 10 minutes. The patient was treated with dimethyl isopropyl azulene ointment every day until she was referred to our hospital. On the 7th day after the injury, she was referred for medical treatment. Upon initial examination, the local findings were mainly erythema that did not disappear with pressure drainage. However, the skin on the dorsum of the foot, the dorsum of the toes, and the plantar aspect of the foot gradually changed into white necrotic tissue, and the local findings did not change except for the sole of the foot (Figure 1C, 1D). On the 10th day after the injury, surgery was performed under general anesthesia. We performed debridement with sequential excision and found necrosis of the MP joint capsule in the big toe. We then performed a dissection of the big toe at the MP joint level. The dorsum of the second and fifth toes, the dorsum of the foot, and the plantar region of the foot showed some dermal bleeding upon sequential excision, but most of the injuries were beyond the dermis. Debridement of the plantar region was performed. Finally, a 12/1000 inch segmental skin graft was taken from the left thigh, and split-thickness skin grafting was performed. The patient was discharged 3 months after the injury (Figure 1E).

Case 2

**Figure 2:**
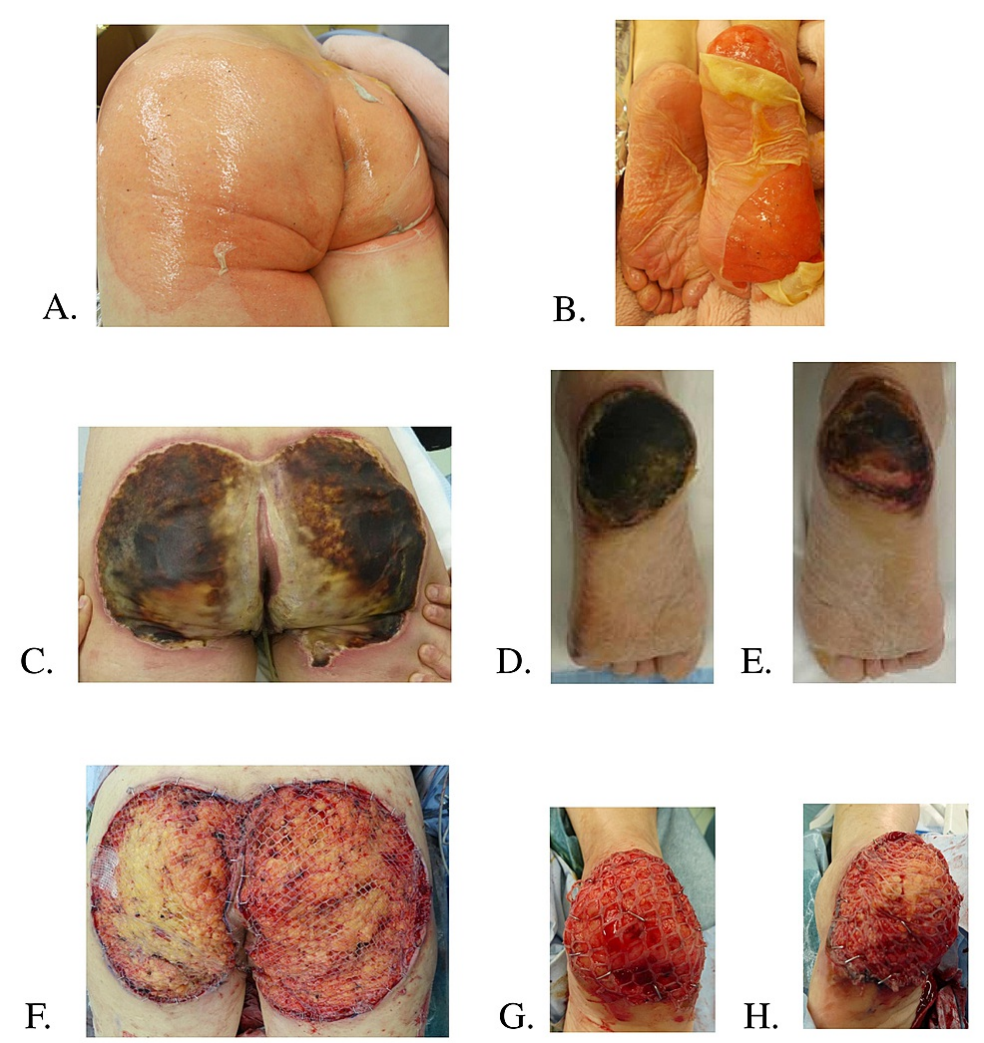
Case 2 (71-year-old woman). (A,B) Day 2 after the burn injury. Ulcerative lesions with partial rupture of the blister cap on both buttocks and bilateral soles are noted. Wound bases are reddish in color. (C,D,E) Day 12 after the injury. This photograph was taken just before surgery. The erythematous areas of the bilateral buttocks are white in the center with necrotic tissue on the outer side. The plantar forefoot of both feet has healed, but the heels of the feet are associated with necrotic tissue. (F,G,H) Day 12 after the injury. Debridement and split-thickness skin grafting were performed under general anesthesia. Bilateral buttocks and heels were debrided by sequential excision, and bleeding from the dermis was observed in a small area of the heel; in most areas, adipose tissue was exposed beyond the dermis. Except for the heels of both soles, epithelialization had already occurred at the time of surgery. Skin grafts were then taken from the left thigh at 12/1000 inches, processed into a triple mesh, and split-thickness skin grafting was performed on both buttocks and both heels.

A 71-year old woman presented with a history of type 2 diabetes and osteoarthritis in both knees. The temperature of the bath was approximately 44°C, so she tried to cool down the water. However, it was difficult for her to move because of knee osteoarthritis. She called out for help to her neighboring family, but they did not take notice of her. She sat there for 2 hours and eventually suffered scald second-degree burns on both buttocks and plantar regions of the feet. After the emergency call, the family cooled the patient with cold water for 30 minutes. At the time of transport to a treatment facility, erythema was the only clinical sign; therefore, she was diagnosed with a superficial dermal burn (Figure 2A, B). The patient was treated with dimethyl isopropyl azulene ointment every day until she was referred to our hospital. However, on the 7th day after the injury, the burn wound deepened, and she was referred for further medical treatment. The burn surface at the time of consultation was deep with 6% total burn surface area (Figure 2C-E). On the 12th day after the injury, debridement and split-thickness skin grafting were performed under general anesthesia. First, bilateral buttocks and heels were debrided by sequential excision, and dermal bleeding was observed in a small area of the heel; however, adipose tissue was exposed beyond the dermis in most areas (Figure 2F-H). Except for both heels, epithelialization had already occurred at the time of surgery. Skin grafts were subsequently taken from the left thigh at 12/1000 inches, processed into a triple mesh, and split-thickness skin grafting was performed on both buttocks and both heels. She was transferred to the primary care hospital on the 20th-day post-injury. Skin grafts were engrafted on the heel and 80% engrafted on the buttocks (photographs of the wound at the time of transfer were not taken).

## Discussion

Comfortable-temperature scald burns were investigated by Moritz [4] using two vectors, “heat source temperature” and “contact time.” They found that the time to onset of irreversible changes in the epidermis was divided by half when the contact temperature increased by 1°C [4]. Suzuki et al. [3] added that these inversely proportional curves reached the critical point at a lower temperature owing to individual factors such as circulatory failure due to compression. An environment in which comfortable-temperature burns are likely to occur is developed by applying a load such as compression or dehydration. In addition, individual factors include decreased activity and paresthesia, specifically from excess alcohol consumption, diabetes, post-cerebral infarction paralysis, and among elderly individuals and infants.

Many Japanese soak in hot water before going to bed to recover from fatigue. As mentioned, many cases of burns of thermal liquid due to comfortable temperature were experienced at the time of bathing in elderly people. Hence, we presented typical cases of experience from the peculiarity of the onset. The water supply temperature was set to approximately 44°C in each case, giving the impression of a high temperature. However, our facility is located in Oita Prefecture, home to Beppu Onsen, one of the largest hot springs in Japan. Therefore, many elderly people set the water temperature at high levels.

In the case of a burn caused by a heat source slightly hotter than the body temperature, the pathogenic mechanism is similar to that of a low-temperature burn, unlike a typical scald burn. Furthermore, a factor that may cause elderly individuals to be easily injured is decreased local blood flow due to dehydration. Sweating associated with bathing, and fainting due to a hot environment is likely to occur among those who bathe. It is proposed that various individual factors such as delayed detection due to sensory paralysis arising from neuropathy and other neurological diseases or metabolic diseases like diabetes, combined with decreased activity due to old age and a history of orthopedic diseases lead to easy injury. According to the 2019 Basic Survey on Living Conditions conducted by the Ministry of Health, Labour and Welfare [5], 59.7% of the respondents were aged 65 years or older, and 33.1% were aged 75 years or older and living with a primary caregiver. In addition, according to the National Institute of Population and Social Security Research [6], the number of households headed by a single person aged 65 or older is expected to increase to 40% by 2040. Considering these findings, a delay in discovery and the time lag until an emergency request are factors considered in the deepening of burns. The abnormally extended contact time with the thermal liquid, combined with lesion degeneration similar to that of low-temperature burns allows the scald to deepen. Furthermore, the temperature of the bathwater does not decrease immediately since the temperature is kept constant by the continuous addition of hot water. This is considered to be one of the causes of deepening scald burns.　

**Figure 3:**
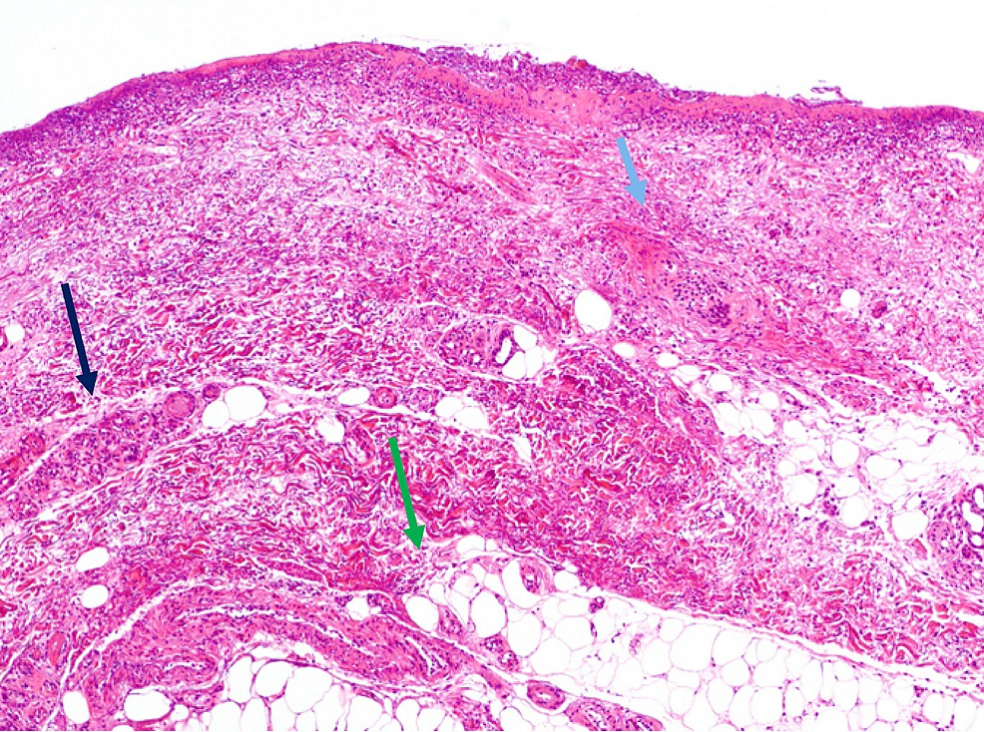
Case 2 Histopathological findings. Biopsy image from the erythematous area of the buttocks (low power field). Findings of total skin necrosis, and necrosis of blood vessels (light blue arrows), sweat glands (black arrows), and fat (green arrows) are present.

Both cases were referred to the hospital about 1-week post-injury. A characteristic of the second case is that in the early stage of injury, erythema with strong redness often occurs. It is speculated that the degree of burn depth may be misdiagnosed as superficial dermal burn (SDB) at the first assessment. In addition, the second case presented with a histopathological evaluation of the erythema site on the buttocks that revealed the necrosis of the blood vessels, sweat glands, and fat tissue, which was a DB finding (Figure 3). Therefore, it is necessary to perform a depth evaluation method, such as a pinprick test or a hair removal method, or laser Doppler blood imaging [7]. However, at the initial treatment stage, the former two evaluation methods were used because they are simple and were preferable for use in non-specialized burn facilities. Generally, if no pain is found in the pinprick test, deep dermal burn (DDB) and DB are suspected, and if it is a hairy part, DB is suspected by plucking without resistance by the hair removal method. However, DDB and DB are difficult to distinguish [8]. Therefore, it is necessary to evaluate the depth of invasion, together with the initial clinical examination and history of injury.

Low-temperature burns during bathing in elderly people are often seen in the heel and buttocks where the load is placed in a sitting position during bathing, as seen in the second case. The same tendency was observed in cases other than the two presented here. Fainting is also likely to occur because of the hot environment, and it is thought that pathogenic mechanisms such as coma blisters are involved [9]. Coma blisters are more likely to occur in the cases of peripheral circulatory disorders or hypoxia. Among elderly people with underlying diseases, peripheral circulatory disorders and swelling were associated with dehydration and hypoxia due to fainting. It is expected that the environment will be prone to the development of coma blisters. Due to such a complex factor, there is a concern that elderly people may sustain low-temperature burns when taking a bath in a liquid at a comfortable temperature.

Finally, treatment was performed. Surgical interventions such as debridement should be performed from an early invasion assessment, rather than conservative treatment, without an early and appropriate assessment, and an appearance-based diagnosis of SDB. Choi et al. [10] reported that early surgical intervention shortened the healing period. However, in these cases, it often takes a certain period of time to receive a referral, since they are judged first if they are suitable for surgery, and treatment intervention is often delayed. After epithelialization, it is desirable to promote early wake-up and rehabilitation to return to daily life as soon as possible.

## Conclusions

We encountered cases of low-temperature burns observed among elderly people while bathing. Many had delayed treatment due to early burn depth assessed as SDB due to the appearance of erythema. This showed the importance of taking the medical history, doubting the possibility of DDB and DB, and performing evaluation methods for in-depth assessment, such as the pinprick test and hair removal method, since they are part of the initial treatment for burns. Considering that the injury occurred during bathing, a combination of factors could easily cause deep burns, such as an injury mechanism similar to that of coma blisters, dehydration in a hot environment, loss of consciousness due to vagal reflexes, and physical disorders such as orthopedic diseases. Moreover, there are social issues that play a role such as nursing care for elderly people and elderly people living alone. Therefore, future studies may focus on these issues to prevent these injuries from occurring.
